# EP08 A case of granulomatosis with polyangiitis complicated by COVID-19: challenges in diagnosis and management

**DOI:** 10.1093/rap/rkaa052.007

**Published:** 2020-11-03

**Authors:** Hanaa Rajabally, Catherine Morley, Shalabh Srivastava

**Affiliations:** South Tyneside and Sunderland NHS Foundation Trust, Sunderland, United Kingdom

## Abstract

**Case report - Introduction:**

Granulomatosis with Polyangiitis (GPA) is a rare small- to medium-vessel vasculitis associated with anti-neutrophil cytoplasmic autoantibody (ANCA). Its multi-systemic features include pulmonary, ear, nose, and throat (ENT), renal, and neurological manifestations. Its incidence is estimated to be 10.2 cases per million population. It is challenging to diagnose when its symptoms are treated in isolation from one another. This case highlights the difficulty in diagnosing GPA in a patient with respiratory symptoms during the Coronavirus Disease 2019 (COVID-19) pandemic and describes the challenges of managing it in the context of a subsequent COVID-19 infection as the mainstay of treatment remains immunosuppression.

**Case report - Case description:**

A 78-year-old female non-smoker with a history of leg ulcers developed a 3-month history of cough and haemoptysis and was treated in primary care for suspected sinus and chest infections. She then presented to Accident and Emergency twice for the same symptoms and was discharged after having her antibiotics changed.

2 weeks later, she presented for the third time with cough, ongoing haemoptysis, conjunctivitis in the right eye, pain over the right side of her head, and discharge from her right ear. She was admitted as she was pyrexical, tachycardic and her CRP was 60. COVID-19 swabs were negative. ENT team recommended IV ceftriaxone and metronidazole for suspected orbital cellulitis. Blood cultures remained negative. CT sinuses with contrast showed right sided thrombosis of transverse sinus and bilateral mastoid effusion of the middle ear. Following neurology review, she was anticoagulated with dalteparin. A day later, she was transferred to the Respiratory ward and dropped her Haemoglobin level to 70. Her chest radiograph showed diffuse alveolar haemorrhage and CT images showed widespread bilateral peri-hilar consolidation.

A rheumatology opinion was sought and vasculitic screen showed ANCA 268, and PR3 >177. Her urinary protein/creatinine ratio was elevated at 90. Rheumatology team confirmed multi-systemic GPA and recommended starting oral Prednisolone 60 mg daily. After the renal team was consulted, she was moved to a side-room and started on IV Methylprednisolone (pulsed with three doses), along with cyclophosphamide and rituximab. Dalteparin was discontinued.

2 days later, she desaturated, and became pyrexical. Repeat COVID-19 swabs were positive.

Three Consultants agreed that Plasma Exchange and Non-Invasive Ventilation (NIV) would be inappropriate. A Do Not Attempt Resuscitation form was signed, and prognosis was discussed with the patient and her 78-year-old husband who requested to visit. Patient deteriorated and unfortunately died 6 days later.

**Case report - Discussion:**

This case is interesting because it highlights the diagnostic challenge of GPA. Retrospectively, it may be noted that doctors persisted in treating suspected infection although the patient continued to deteriorate. However, a diagnosis should be re-considered if the patient does not respond to treatment and it is important to consider vasculitis as a cause of haemoptysis.

Anticoagulation was started since the benefits were considered to outweigh the risks as her haemoptysis was of small volume. The patient soon developed pulmonary haemorrhage, so the risks of anticoagulation should not be underestimated in vasculitis.

The Rheumatology team’s cautious approach to immunosuppression was in stark contrast to the renal team’s aggressive approach. The Renal team believed that concerns about protecting the patient from COVID-19 when she was negative from this infection should not take precedence over appropriate immunosuppression from a potentially fatal vasculitis.

The patient was admitted at the start of the COVID-19 pandemic and was negative for COVID-19 on admission. She was nursed in a bay on the Respiratory ward where she later became COVID-19 positive. This raises questions about whether the earlier test was a false negative result or whether her infection was hospital-acquired. Infection control guidelines were changing rapidly at the start of the COVID-19 pandemic.

The decision to avoid plasma exchange was based on the findings of the PEXIVAS trial. NIV was avoided as it required a full-face mask to minimize particle dispersion but would pose an asphyxiation risk as patient was coughing up blood.

Finally, the team learnt to be flexible in these extraordinary circumstances when dealing with the end-of-life decisions of the COVID-19 positive patient. Although her husband was a vulnerable person because of his age, he was given the opportunity to visit while wearing Personal Protective Equipment and agreed to self-isolate for two weeks.

**Case report - Key learning points:**

This case helped me appreciate the complexity of deciding to immunosuppress an already severely ill patient in the context of the COVID-19 pandemic. I recognised that the patient had a poor prognosis with or without immunosuppression and our role as healthcare professionals was to give her the best chance of recovery. The conference will allow me to interact with other colleagues and discuss what they would do in this situation as our Rheumatology and Renal teams had different approaches.

After further reading on false negative results, we found that Johns Hopkins researchers found that testing people for SARS-CoV2 too early in the course of infection is likely to result in a false negative test even though they may eventually test positive for the virus.

I have also learnt about the PEXIVAS trial which found that the addition of plasma exchange to standard therapy does not reduce the risk for all-cause mortality among patients with severe ANCA-associated vasculitis. Moreover, a reduced-dose regimen of glucocorticoids is non-inferior to a standard-dose protocol, while reducing the risk for serious infections.

Diffuse alveolar haemorrhage (DAH) is not treatable with arterial embolization or bronchoscopic methods due to the diffuse nature of the bleeding. Extracorporeal membrane oxygenation (ECMO) has been used to support patients with DAH but the use of ECMO is controversial due to the need for anticoagulation.

The conference will help me deepen my understanding of epidemic rheumatology which will be useful for my clinical practice going forward, especially if there is a second wave of the COVID-19 pandemic. I am keen to use this event to engage with other clinicians on immunosuppression in the context of infection so that I may confidently manage similarly complex cases in the future.

